# Hypochlorous Acid as a Potential Wound Care Agent: Part II. Stabilized Hypochlorous Acid: Its Role in Decreasing Tissue Bacterial Bioburden and Overcoming the Inhibition of Infection on Wound Healing

**Published:** 2007-04-11

**Authors:** Martin C. Robson, Wyatt G. Payne, Francis Ko, Marni Mentis, Guillermo Donati, Susan M. Shafii, Susan Culverhouse, Lu Wang, Behzad Khosrovi, Ramin Najafi, Diane M. Cooper, Mansour Bassiri

**Affiliations:** a Institute for Tissue Regeneration, Repair and Rehabilitation, Bay Pines, FL; b University of South Florida, Tampa, FL; c NovaBay Pharmaceuticals, Inc, Emeryville, CA; d Healthpoint Ltd, Forth Worth, TX

## Abstract

**Background:** A topical antimicrobial that can decrease the bacterial bioburden of chronic wounds without impairing the wound's ability to heal is a therapeutic imperative. A stabilized form of hypochlorous acid (NVC-101) has been demonstrated in vitro and in standard toxicity testing to possess properties that could fulfill these criteria. **Materials and Methods:** Using a standard rodent model of a chronically infected granulating wound, various preparations of NVC-101 and multiple treatment regimens were investigated to evaluate the role of NVC-101 in decreasing tissue bacterial bioburden and overcoming the inhibition of infection on wound healing. Quantitative bacteriology of tissue biopsies and wound healing trajectories were used to compare the various NVC-101 preparations and regimens to saline-treated negative controls and silver sulfadiazine–treated positive controls. **Results:** NVC-101 at 0.01% hypochlorous acid with a pH of 3.5 to 4.0 proved to be an effective topical antimicrobial. It was most effective when used for a brief period (15–30 minutes), and followed with another application. Possibly this was due to its rapid neutralization in the wound bed environment. Although not as effective at decreasing the tissue bacterial bioburden as silver sulfadiazine, NVC-101 was associated with improved wound closure. **Conclusions:** This stabilized form of hypochlorous acid (NVC-101) could have potential application as an antimicrobial wound irrigation and treatment solution if its effective pH range can be maintained in the clinical situation. NVC-101 solution was equally effective at pH 3.5 or 4.0 and more efficient soon after its application. As opposed to other antimicrobials investigated in this animal model, NVC-101 controls the tissue bacterial bioburden without inhibiting the wound healing process.

Wound healing is the end result of a series of interrelated cellular processes initiated by humoral factors such as cytokine growth factors.^1^ These cellular processes are inhibited by a large tissue bacterial bioburden.^2^ The cytokines and growth factors are also degraded by bacteria.^3^ The level of tissue bacterial bioburden has been shown in multiple studies to be more than 10^5^ or at least 1 × 10^6^ bacteria per gram of tissue.^4^,^5^ Such high levels of tissue bacteria can be present without clinical signs of infection, and when present can deleteriously affect wound healing.^6^

Attempts at controlling the tissue bacterial bioburden have been difficult. Systemically administered antibiotics do not effectively decrease the level of bacteria in a chronic granulating wound.^7^ Therefore, topical antimicrobials or temporary biologic dressings have been the methods of choice.^4^,^8^ Topical use of antibiotics that are used effectively systemically for purposes other than wound infection is discouraged because of an increased risk for developing allergies or the potential for bacteria to develop resistance to the drug.^9^ Antiseptics and nonantibiotic antimicrobials such as povidone-iodine, silver sulfadiazine, or mafenide acetate cream have been demonstrated to be cytotoxic to the cellular components of wound healing.^10^–^12^

Stabilized hypochlorous acid (NVC-101) prepared by the addition of sodium hypochlorite to a solution of sodium chloride in sterile water followed by addition of a solution of hydrochloric acid and maintained at a pH between 3.5 and 5 has been demonstrated to have excellent in vitro antibacterial properties. Its potential limitation is the requirement to maintain its narrow pH range in the clinical wound environment.

The purpose of the studies reported here was to evaluate various concentrations of stabilized hypochlorous acid (NVC-101) topically administered to an experimental chronic infected granulating wound at different pHs and with different treatment regimens. The effects evaluated were the ability of NVC-101 to control the tissue bacterial bioburden and the ability of the agent to overcome the inhibition of wound healing caused by infection.

## MATERIALS AND METHODS

### Reagents

The various NVC-101 preparations ranging in concentrations from 0.01% to 0.02% and from pH 3.5 to 4.5 were provided by NovaBay Pharmaceuticals, Emeryville, Calif. In brief, the stabilized HOCl was prepared in 150 mM NaCl by acidifying reagent-grade NaOCl to the pH of 3.5 to 4.5 with dilute HCl. The concentration of active chlorine species ([HOCl]_T_ = [HOCl] + [Cl_2_] + [Cl_3_] + [OCl^−^]) in 0.9% saline was determined by converting all the active chlorine species to OCl^−^ with 0.1 M NaOH and measuring the concentration of OCl^−^ spectrophotometrically at 292 nm using a molar absorbtivity of 362 M^−1^ cm^−1^.^13^

### Microbiological methods

*Escherichia coli* (ATCC 25922) was purchased from the American Type Culture Collection, and grown and propagated according to the ATCC recommendations. Bacteria for use in the animal model were obtained from fresh 18-hour broth culture, and inoculum size was confirmed by back-plating.

### Animal model of chronic granulating wound

Chronic granulating wounds were prepared as previously described.^7^,^14^–^16^ Male Sprague-Dawley rats weighing 300 to 350 g were acclimated in the facility for a week before use. Under intraperitoneal pentobarbital (Nembutal) anesthesia (35 mg/kg), the rat dorsum was shaved and depilated. A full-thickness dorsal burn measuring 30 cm^2^ was created by immersing in boiling water. Infected groups were seeded with 5 × 10^9^ CFU of *E. coli* (ATCC 25922) after the rats had been allowed to cool for 15 min.^16^

Animals were individually caged and given food and water ad libitum. Uninfected animals were kept in a physically separate facility. All experiments were conducted in accordance with the American Care and Use Committee at the Department of Veterans Affairs Medical Center, Bay Pines, Fla.

Five days after burning, the eschar was excised from anesthetized animals, resulting in a chronic granulating wound. Histological characterization of this wound with comparison to a human granulating wound has previously been performed.^7^

### Treatment groups

Two different experiments using multiple treatment regimens were performed. In experiment 1, 45 rats were divided into 9 groups of 5 animals each. The groups were treated as follows: group I served as uninfected controls and received no inoculation of bacteria. Following escharectomy, these rats were treated with a saline (0.9% NaCl)-soaked gauze dressing, which was changed every 24 hours. Group II was an infected control and was inoculated, as were groups III to VIII. After escharectomy, the rats in group II were treated with daily changes of saline-soaked gauze dressing. Group III animals had their escharectomized infected wounds treated with gauze dressing saturated with 0.01% NVC-101, pH 3.5, changed every 24 hours. Groups IV and IVb were treated identically. Animals in these 2 groups had their escharectomized infected wounds treated with a dressing soaked with 0.01% NVC-101, pH 3.5, which remained in place for 30 minutes and then was replaced with a dressing soaked in 0.9% NaCl for 23.5 hours. This regimen was repeated every 24 hours. Group V received similar treatment as group III except that the 0.01% NVC-101 had a pH of 4.0. The regimen for group VI was similar to groups III and V except that the pH was adjusted to 4.5. Group VII was treated similarly to group III except that the concentration of NVC-101 was increased 0.02%, with the pH at 3.5. Finally, group VIII animals were treated with 1% silver sulfadiazine cream (Silvadene) without dressing and changed every 24 hours. The moist gauze dressings in groups I to VII were covered with one layer of petrolatum-impregnated gauze (Adaptic) and then covered with Coban dressing. A summary of animal treatment groups is depicted in Table 1.

Following evaluation of the results of experiment 1, in vitro modifications of techniques were investigated. It was decided that wiping off the wound following an initial application of stabilized hypochlorous acid and then replacing it may have added benefits (data not presented).

Experiment 2 consisted of 8 groups of 5 animals each. Group I served as the infected control and escharectomized infected wounds were treated with 0.9% NaCl–soaked dressing changed every 24 hours. Group II was treated with a gauze soaked in 0.01% NVC-101, pH 3.5, for 15 minutes, followed by gentle atraumatic wiping of the wound, and then treated by another application of 0.01% NVC-101, pH 3.5, for 23.75 hours. This regimen was repeated every 24 hours. Group III was treated the same as group II except that the pH of NVC-101 solution was adjusted to pH 4.0. The regimen for group IV was identical to group III using the pH 4.0 solution as the first application. However, after the gentle wiping, 0.9% NaCl was substituted for the remaining 23.75 hours instead of a repeat application of NVC-101. Group V had normal saline (0.9% NaCl) applied on the first dressing for 15 minutes, followed by wiping, and then another saline-soaked dressing for 23.75 hours. This was repeated every 24 hours. Group VI was treated identical to group II and had a 15-minute application of 0.01% NVC-101, pH 3.5, followed by wiping, but then followed by a gauze dressing soaked with 0.01% NVC-101, pH 3.5, left in place for 47.75 hours. This was repeated every 48 hours. Group VII mimicked group VI except that the second dressing consisted of a saline-soaked sponge for 47.75 hours. Finally, group VIII animals were treated after escharectomy with a 0.9% NaCl–soaked dressing for 30 minutes, followed by 23.5 hours of a second saline-soaked dressing. No gentle wiping was interspersed between dressings in group VIII. A summary of the animal treatment groups in experiment 2 is depicted in Table 2.

### Animal procedures

In experiment 1, rats were premedicated with buprinorphine (0.1 mg/kg) and anesthetized with halothane inhalation on postescharectomy days 4, 8, 12, 16, and 20. Any dried exudates that formed were atraumatically removed. Wounds were biopsied for quantitative bacteriology on the day of escharectomy (day 0) and on each of the days of reanesthesia according to the methods described by Heggers and Robson.^5^ The wound surface was cleaned with 70% isopropyl alcohol prior to biopsy to exclude surface contamination. Biopsies were aseptically weighed, homogenized, serially diluted, and back-plated onto nonselective media. Bacterial counts were completed after 48 hours' incubation and expressed as colony-forming units (CFU) per gram of tissue.^5^

While the rats were anesthetized for the wound biopsies, outlines of the wounds were traced onto acetate sheets, and area calculations were performed using computerized digital planimetry (Sigma Scan Jandel Scientific, Corte Madera, CA). Care was taken only to record the perimeter of the wound that represented the advancing full-thickness margin rather than the edge of any advancing epithelium. This avoided the small component of advancement provided by the smooth, pink, translucent, hairless neoepithelium.^16^ All animals were weighed at the time of biopsy and wound measurement.

The animals were sacrificed by Nembutal overdose and bilateral thoracotomies when the wound had completely healed or decreased to less than 10% of its original area. Haywood et al demonstrated that measurement of very small wounds by manual tracing introduced significant systematic error and found that wounds followed past this point remained static for prolonged periods of time.^17^

The animals in experiment 2 had the same procedures performed as those in experiment 1 except they were performed at different time points, that is, days 0, 2, 4, 7, 9, 11, and 14, with the final wound size recorded on day 16. The time points were chosen to capture earlier time points and more frequent changes in the wound size and bacteriology.

### Statistical analysis

Mean bacterial counts for each group of animals in both experiments were determined and expressed as CFU/g of tissue. These values were compared for each experiment using a one-way analysis of variance. Post hoc analyses of differences between groups were carried out using Tukey's test (all pairs, multiple-comparison test), with *P* < .05 considered significant. Sigma Stat statistical software (Jandel Scientific, Corte Madera, CA) was used for data analysis.

Serial wound area measurements were plotted against time. For each animal's data, a Gompertz equation was fitted (typical *r*^2^ = 0.85).^18^ Using this approach, a best-fit curve was generated for each group. Comparison between groups was performed using life table analyses and the Wilcoxon rank test. These statistical analyses were performed using SAS^19^ and BMDP^20^ packages on a personal computer.

## RESULTS

### Quantitative bacteriology

**Figure 1:**
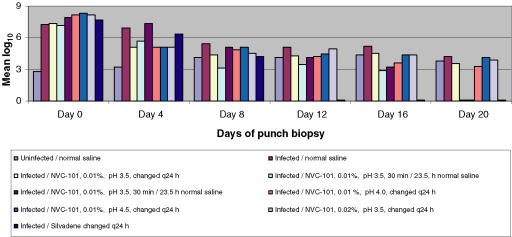
Depiction of the various groups in experiment 1 demonstrating the superiority of 30 minutes of NVC-101 application followed by another dressing for 23.5 hours over other regimens of hypochlorous acid. 1% silver sulfadiazine cream (Silvadene) was the most effective of all agents tested at decreasing the tissue bacterial bioburden.

Quantitative bacteriology of the chronic granulating wounds treated with various formulations of stabilized HOCl (NVC-101) or Silvadene were determined. The mean bacterial counts for each biopsy day in experiment 1 are depicted in Table 3. Plots of mean log_10_ versus time for the various treated groups in experiment 1 are depicted in Figure 1 with the statistical comparisons.

**Figure 2:**
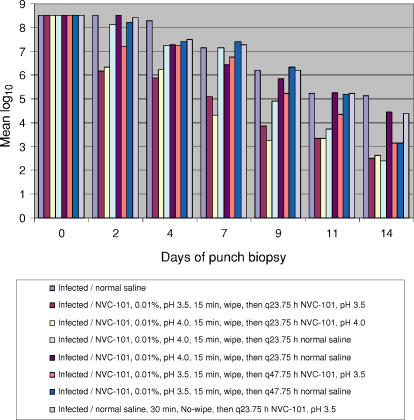
Depiction of the various groups in experiment 2 demonstrating the superiority at decreasing bacterial counts of NVC-101 applied for 15 minutes, followed by gentle atraumatic wiping then 23.75 hours of a second dressing of NVC-101. There was essentially no difference in the effects with pH 3.5 and 4.0.

It is clear that Silvadene was the best topical antimicrobial at decreasing the tissue bacterial burden. 0.01% NVC-101, pH 3.5, applied for 30 minutes and then removed from the wound proved to be the next most effective regimen for decreasing the bacterial load in experiment 1. This regimen was used in both groups IV and IVb and the results were similar (Table 3 and Fig 1). The bacterial data from experiment 2 are also depicted in Table 4. Plots of mean log_10_ versus time for the various groups in experiment 2 are depicted in Figure 2 with statistical comparisons. Experiment 2 greatly expanded the knowledge of dosing regimen for NVC-101. Experiment 2 looked more carefully at the earlier, more frequent time points. Three regimens in experiment 2 were as good as or better than the best regimen in experiment 1 at decreasing the tissue bacterial burden. Groups II, III, and IV all had counts less than 10^3^ CFU/g of tissue by day 14. In groups II and III, which had essentially the same treatment regimens, the bacterial counts decreased more rapidly than in group IV. For these groups (II and III), the regimen consisted of NVC-101 being placed on the wound for 15 minutes, atraumatically wiped off, and then reapplied for 23.75 hours. The only difference between the treatments for groups II and III was the pH of NVC-101. No significant differences were seen between pH 3.5 and pH 4.0 (Table 4 and Fig 2).

### Body weights

There was an equivalent gain in body weight among all groups during the period of study, with no significant variations among the groups in either experiment 1 or experiment 2

### Wound area

**Figure 3:**
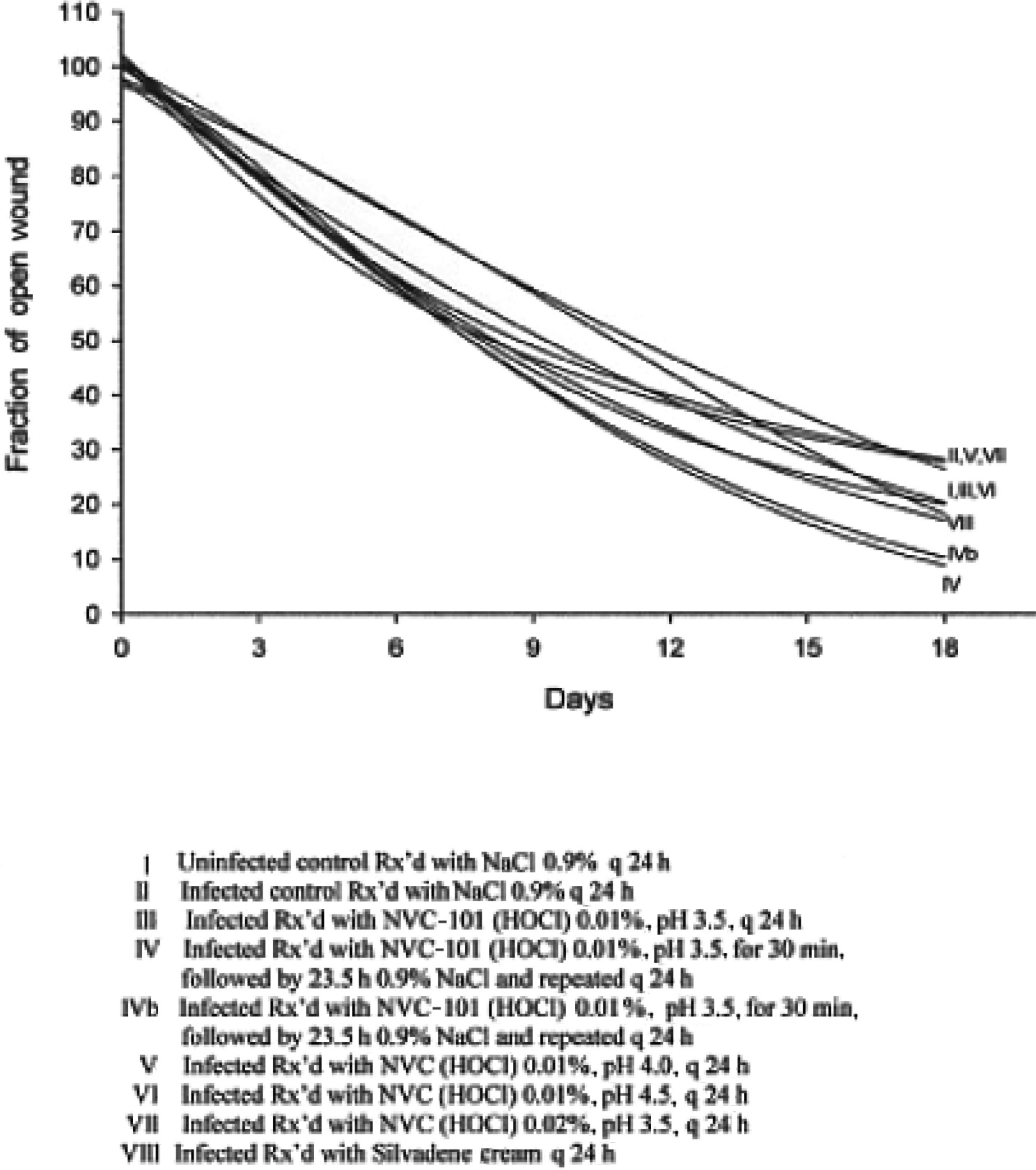
Wound healing trajectories from experiment 1, demonstrating faster healing for groups IV and IVb treated with a short application of NVC-101 followed by a second dressing of normal saline. These 2 groups had a more favorable wound healing trajectory than did group VIII treated with 1% silver sulfadiazine cream (Silvadene).

**Figure 4:**
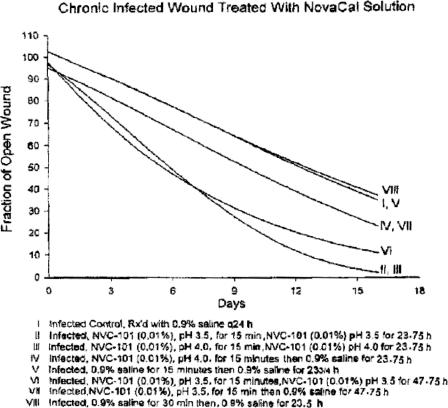
Wound healing trajectories from experiment 2, demonstrating the superiority of groups II and III, which consisted of a brief application of NVC-101, followed by gentle wiping, and a second application of NVC-101 over other regimens. The 2 groups with the fastest healing were also the most effective at controlling the tissue bacterial bioburden.

Best-fit healing curves demonstrated that none of the treatment regimens resulted in the area of the wound increasing in size (Figs 3 and 4).

Infected control animals (group II in experiment 1, group I in experiment 2) retarded healing as compared to the noninfected controls (group I in experiment 1). Healing curves for groups IV and IVb in experiment 1 demonstrated statistically significant increases in reduction in the fraction of open wounds when compared to groups I, III, V, and VIII (*P* < .05) and groups II, V, and VII (*P* < .01) (Fig 3). Groups II, V, and VII demonstrated a slower trajectory than all other groups, also statistically significant (*P* < .05) (Fig 3).

In experiment 2, healing curves for groups II and III demonstrated statistically significant larger reductions in the fraction of open wounds when compared to groups IV, VI, and VII (*P* < 0.05) and groups I, V, and VIII (*P* < .01) (Fig 4). Groups I, V, and VIII demonstrated a slower healing trajectory than all other groups, which was also statistically significant (*P* < .05).

## DISCUSSION

Because of the deleterious effect of a high tissue bacterial burden on the process of wound healing, an effectual antimicrobial agent becomes a therapeutic imperative. Such an agent should be effective as a topical preparation, yet not to be cytotoxic to the cells involved in the wound healing process.^21^ Stabilized hypochlorous acid, as tested in the 2 experiments reported, may prove to be such an agent. Its in vitro antibacterial properties and tissue safety profile suggest its potential as a wound care agent.^13^ However, it is likely rapidly neutralized in the wound environment.

In experiment 1, Silvadene was, as expected, the most effective antibacterial. However, Silvadene was not as effective at promoting wound closure as were two of the NVC-101 regimens (IV and IVb) (Fig 3). The healing that occurred with Silvadene was probably due to elimination of the tissue bacterial load (Table 3). The reason the wounds did not totally heal or exceed that with groups IV and IVb is because of the known cytotoxic properties of Silvadene.^11^,^12^

From a review of the quantitative bacteriology data from both experiments, it is clear that a brief application of NVC-101, followed by a second dressing change is better than a single application of NVC-101 left in place for 24 hours (group III, experiment 1) (Tables 3 and 4).^21^ When the second dressing is again NVC-101 (groups II and III from experiment 2), the rate of bacterial reduction is faster than when the initial application of NVC-101 is followed by normal saline (groups IV and IVb in experiment 1, or group IV in experiment 2) (Table 3 and 4, and Figs 1 and 2). There was no apparent difference in the wound healing trajectory whether the second dressing contained NVC-101 or saline (Figs 3 and 4).

It is clear that the effect of NVC-101 on bacteria occurs in a short period of time after application. Possibly leaving NVC-101 in place for 24 hours stimulates greater plasma or serum response to inflammatory stimuli and that plasma milieu allowed bacterial growth over time. This is not unlike suggestions from Fleming's classic article of 1919.^21^ Therefore, it may be useful to use NVC-101 for a short duration of time. The initial bacterial kill by NVC-101 appeared sufficient not to allow regeneration of bacteria when replaced by saline (groups IV and IVb, experiment 1). The antibacterial effect was obviously due to NVC-101, since when only saline was used in 1 or 2 applications, the bacterial kill was less (group II, experiment 1; groups V and VIII, experiment 2) (Tables 3 and 4, Figs 1 and 2).

The differences between pH 3.5 and 4.0 were not detectable. However, when the pH of NVC-101 was raised to 4.5, the control of the tissue bacterial burden seemed slightly less effective, with a slower wound healing trajectory. Therefore, it appears that a pH of 3.5 or 4.0 may be more useful. However, these differences may not be significant.

The role of the atraumatic wiping between dressing applications is not entirely clear. If NVC-101 kills bacteria immediately or soon after initial contact, then the gentle wiping may remove the devitalized bacteria and any possible debris, allowing the second application of NVC-101 to be in immediate contact with any remaining viable bacteria. This may explain the faster decrease in tissue bacterial levels seen in groups II and III in experiment 2.

In conclusion, the pilot in vivo study results for the 2 experiments indicate that the stabilized form of hypochlorous acid (NVC-101) is equally effective at pH 3.5 or 4.0 and more effective soon after its application. As opposed to other antimicrobials investigated in this animal model, NVC-101 controls the tissue bacterial bioburden without inhibiting the wound healing process.

## Figures and Tables

**Table 1:** Summary of treatment groups of rats in experiment 1

Group*	**Treatment**
I	Uninfected/normal saline
II	Infected/normal saline
III	Infected/NVC-101, 0.01%, pH 3.5, changed q24 h
IV	Infected/NVC-101, 0.01%, pH 3.5, 30 min/23.5 h normal saline
IVb	Infected/NVC-101, 0.01%, pH 3.5, 30 min/23.5 h normal saline
V	Infected/NVC-101, 0.01%, pH 4.0, changed q24 h
VI	Infected/NVC-101, 0.01%, pH 4.5, changed q24 h
VII	Infected/NVC-101, 0.02%, pH 3.5, changed q24 h
VIII	Infected/Silvadene changed q24 h

*Five animals per group.

**Table 2:** Summary of treatment/group of rats in experiment 2

Group*	**Treatment**
I	Infected/normal saline
II	Infected/NVC-101, 0.01%, pH 3.5, 15 min, wipe, then q23.75 h NVC-101, pH 3.5
III	Infected/NVC-101, 0.01%, pH 4.0, 15 min, wipe, then q23.75 h NVC-101, pH 4.0
IV	Infected/NVC-101, 0.01%, pH 4.0, 15 min, wipe, then q23.75 h normal saline
V	Infected/normal saline, 15 min, wipe, then q23.75 h normal saline
VI	Infected/NVC-101, 0.01%, pH 3.5, 15 min, wipe, then q47.75 h NVC-101, pH 3.5
VII	Infected/NVC-101, 0.01%, pH 3.5, 15 min, wipe, then q47.75 h normal saline
VIII	Infected/normal saline, 30 min, no wipe, then q23.75 h NVC-101, pH 3.5

*Five animals per group.

**Table 3:** Summary of mean bacterial counts for each treated group in experiment 1

	**Days of punch biopsy postescharectomy (mean CFU/g)**					
Group*	**Day 0**	**Day 4**	**Day 8**	**Day 12**	**Day 16**	**Day 20**
I	8.4 × 10^2^	2.48 × 10^3^	1.49 × 10^4^	1.63 × 10^4^	3.71 × 10^4^	8.04 × 10^3^
II	2.92 × 10^7^	8.96 × 10^6^	4.88 × 10^5^	1.39 × 10^5^	1.68 × 10^5^	2.12 × 10^4^
III	3.12 × 10^7^	1.39 × 10^5^	3.6 × 10^4^	3.24 × 10^4^	5.28 × 10^4^	5.16 × 10^3^
IV	2.42 × 10^7^	1.72 × 10^5^	1.31 × 10^3^	4.92 × 10^3^	8.8 × 10^2^	1.0 × 10^1^
IVb	9.52 × 10^7^	3.36 × 10^7^	1.28 × 10^5^	1.51 × 10^4^	2.05 × 10^3^	1.5 × 10^2^
V	1.56 × 10^8^	1.37 × 10^5^	8.6 × 10^4^	2.05 × 10^4^	6.36 × 10^3^	3.12 × 10^3^
VI	2.45 × 10^7^	1.44 × 10^5^	1.25 × 10^5^	6.14 × 10^4^	3.56 × 10^4^	1.38 × 10^4^
VII	2.01 × 10^8^	1.45 × 10^5^	5.8 × 10^4^	9.75 × 10^4^	3.89 × 10^4^	9.7 × 10^3^
VIII	6.52 × 10^7^	3.75 × 10^6^	2.4 × 10^4^	NG†	NG	NG

*Five animals per group.

^†^NG indicates no growth.

**Table 4:** Summary of mean bacterial counts for each treated group in experiment 2

	**Days of punch biopsy postescharectomy (mean CFU/g)**						
Group*	**Day 0**	**Day 2**	**Day 4**	**Day 7**	**Day 9**	**Day 11**	**Day 14**
I	5.0 × 10^8^	5.0 × 10^8^	1.27 × 10^8^	1.4 × 10^7^	1.85 × 10^6^	2.32 × 10^5^	1.41 × 10^5^
II	5.0 × 10^8^	1.59 × 10^6^	8.74 × 10^5^	1.03 × 10^5^	8.8 × 10^3^	3.48 × 10^3^	5.0 × 10^2^
III	5.0 × 10^8^	3.24 × 10^6^	2.51 × 10^6^	3.12 × 10^4^	2.54 × 10^3^	3.48 × 10^3^	6.4 × 10^2^
IV	5.0 × 10^8^	1.12 × 10^8^	2.31 × 10^7^	1.37 × 10^7^	9.16 × 10^4^	7.28 × 10^3^	4.0 × 10^2^
V	5.0 × 10^8^	5.0 × 10^8^	2.78 × 10^7^	4.38 × 10^6^	8.53 × 10^5^	2.53 × 10^5^	4.4 × 10^4^
VI	5.0 × 10^8^	2.01 × 10^7^	2.60 × 10^7^	7.74 × 10^6^	2.24 × 10^5^	3.68 × 10^4^	1.60 × 10^3^
VII	5.0 × 10^8^	2.17 × 10^8^	4.06 × 10^7^	4.06 × 10^7^	3.48 × 10^6^	1.95 × 10^5^	1.56 × 10^3^
VIII	5.0 × 10^8^	4.01 × 10^8^	5.24 × 10^7^	2.72 × 10^7^	1.58 × 10^6^	2.42 × 10^5^	4.04 × 10^4^

*Five animals per group.
